# Pulmonary Artery Vasoconstriction Due to Bronchial Obstruction

**DOI:** 10.5334/jbsr.2761

**Published:** 2022-04-28

**Authors:** Thomas Saliba, Hanna Salame, Denis Tack

**Affiliations:** 1 Epicura Hospital, BE

**Keywords:** pulmonary artery vasoconstriction, bronchial obstruction, CTPA, mucoid impaction, pulmonary vein opacification

## Abstract

Pulmonary artery (PA) vasoconstriction resulting from pulmonary ventilation/perfusion imbalance is infrequently detected with computed tomography (CT). An 85-year-old woman presented to the emergency room with dyspnea and desaturation, despite oxygen supply. A CT pulmonary angiography (CTPA) revealed massive central bronchial mucoid impaction in all but the right upper bronchus. Only the right upper pulmonary veins were opacified, which we speculate was linked to the central bronchial obstruction, with identical distributions, through vasoconstriction of the corresponding PAs.

**Teaching Point:** This case demonstrates the unusual imagery-physiology correlation of pulmonary artery vasoconstriction that cannot normally be detected by CTPA.

## Introduction

Hypoxic pulmonary vasoconstriction (HPV) is a pulmonary artery (PA) homeostatic reflex that, responding to alveolar hypoxia, diverts blood flow to better oxygenated areas of the lung [1]. HPV is stimulated by hypoxia around the pulmonary arteriole, causing vasoconstriction [2]. Vasoconstriction improves ventilation/perfusion matching, reducing shunting and avoiding hypoxia, resolving once the insult has passed [1]. Classically, HPV occurs due to collapse, pneumonia, COPD, or asthma [1].

We present the case of an 85-year-old female in whom the CT pulmonary angiography (CTPA) demonstrated severe pulmonary vasoconstriction secondary to hypoxia resulting from bronchial mucus impaction.

## Case History

An 85-year-old female was referred from the nursing home with pyrexia, dyspnea, and a saturation of 65%, despite receiving oxygen. The patient’s history was positive for myocardial infarct, stroke, epilepsy, and dementia. In the emergency department the patient remained hypoxic and dyspneic, with a saturation of 68% in ambient air and rhonchi upon auscultation. Her D-dimers were 1370.

**Figure 1:**
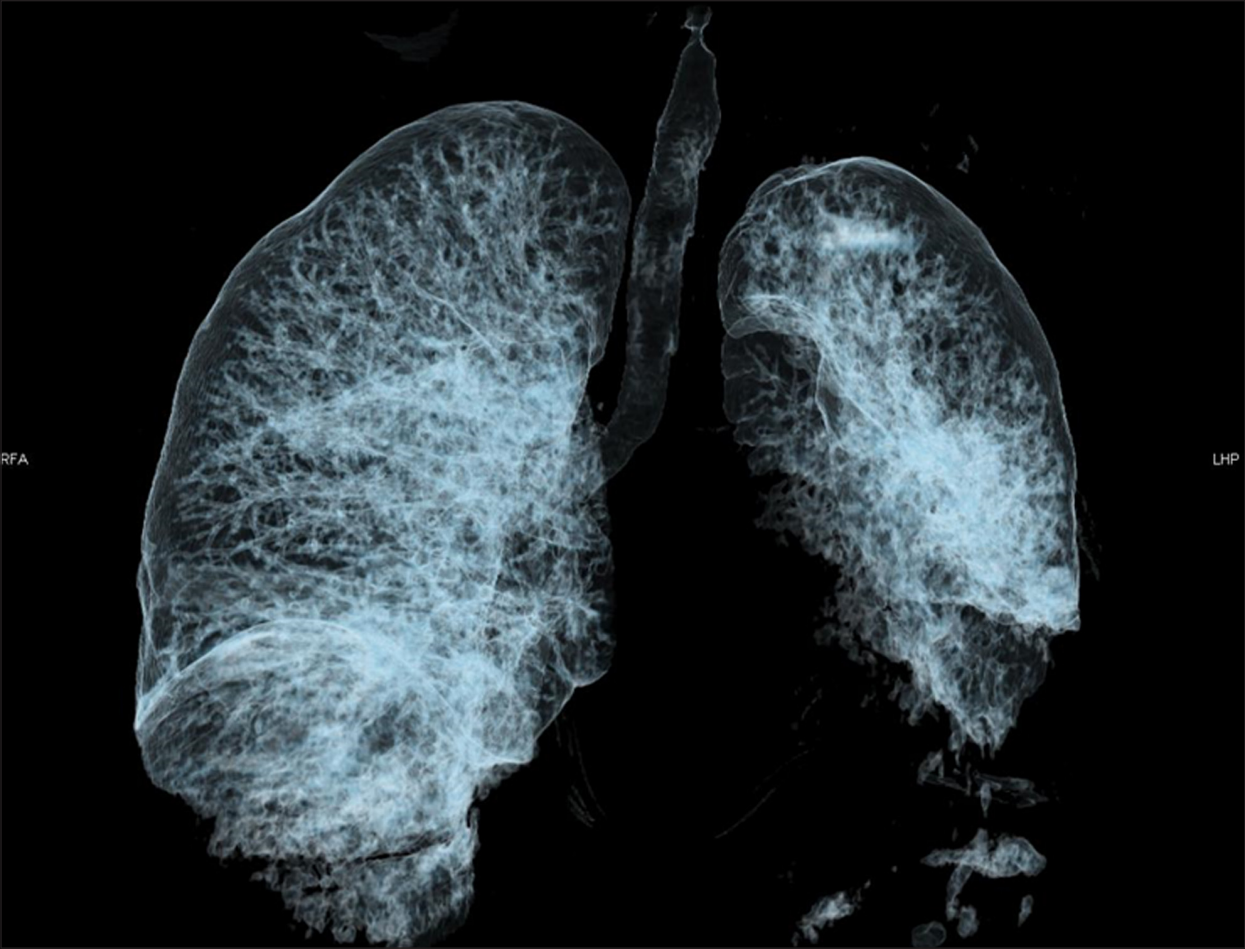
Volume rendering 3D reformat of the airways showing complete obstruction of the non-visible left mainstem bronchus. The air visible in the left lung results from the aerated collapse of the left upper lobe. The left lower lobe suffered non-aerated collapse and thus is not visible.

**Figure 2:**
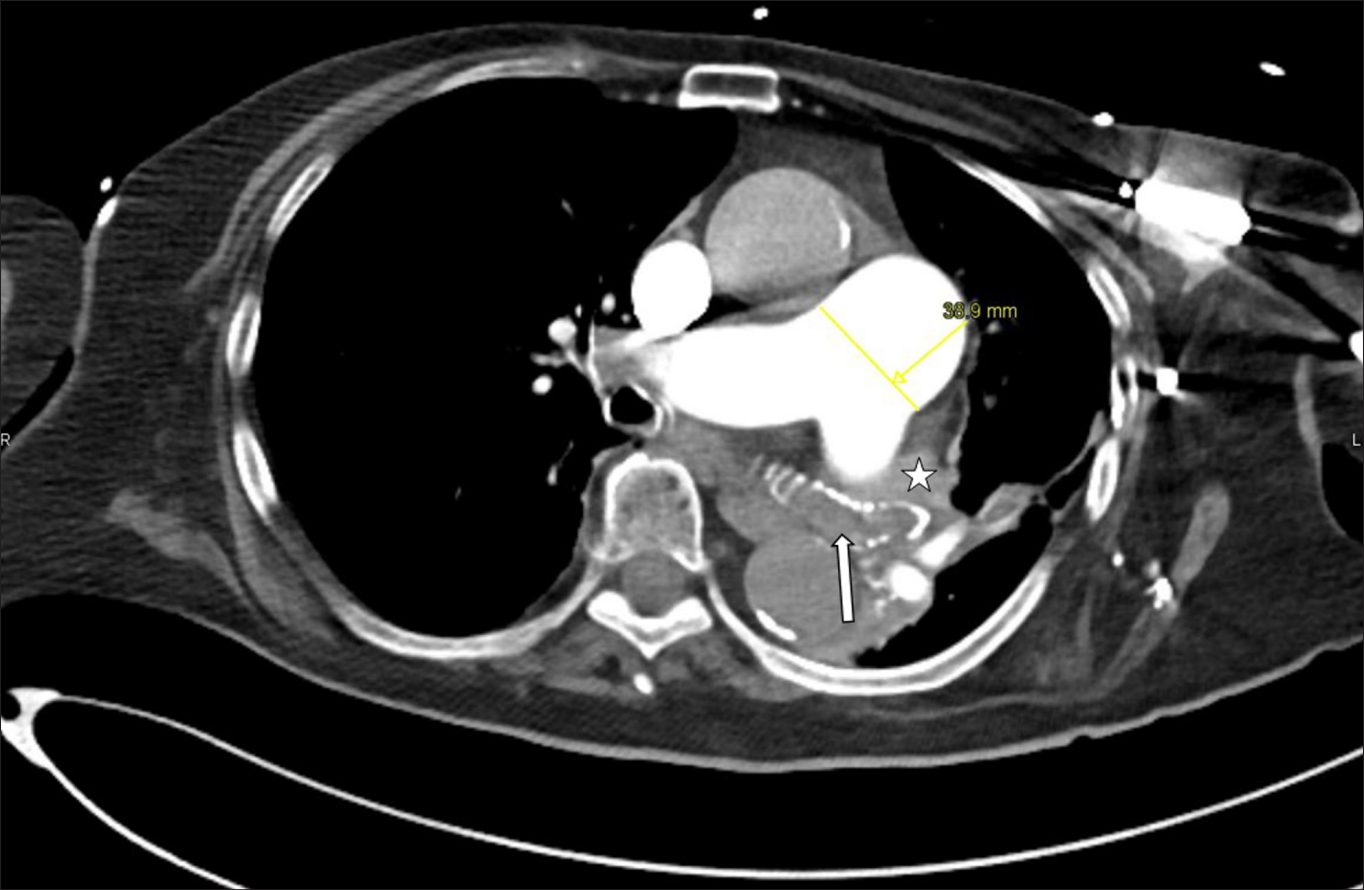
Axial CT of the pulmonary trunk in mediastinal window. Central bronchi on the left side are outlined by calcifications of bronchial walls cartilage and are filled with mucous (white arrow). The left superior pulmonary vein is not opacified (star). The pulmonary trunk diameter is 38.9 mm (normal being ≤27 mm in females), likely because of PA hypertension.

**Figure 3:**
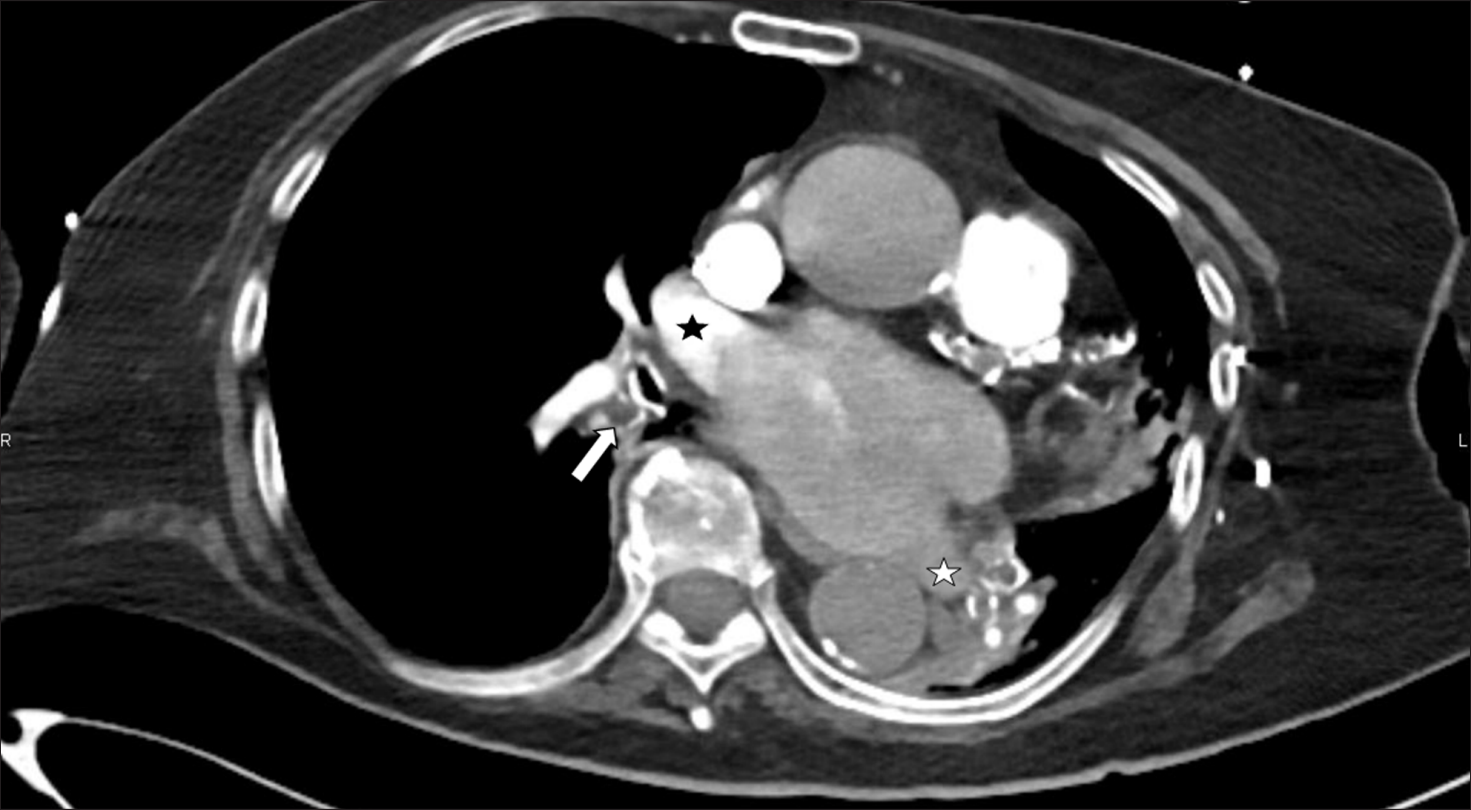
Axial CT of the right lower lobe bronchus that is filled with mucus (arrow), also showing unusual lack of opacification of the left atrium, where only the right superior pulmonary vein is opacified (black star). The left inferior pulmonary vein is not opacified (white star).

**Figure 4:**
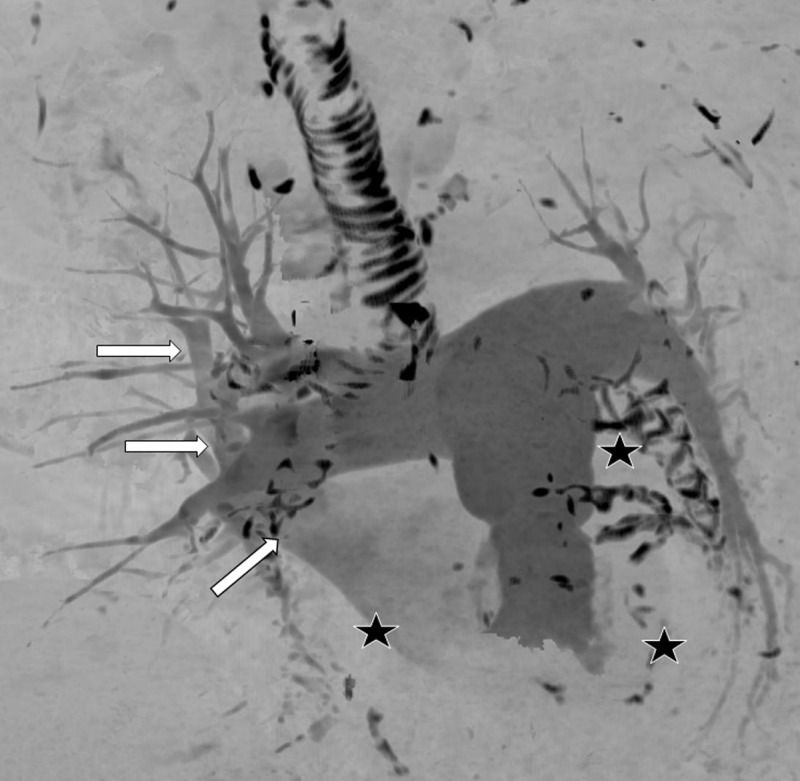
3D maximum intensity projection of all the pulmonary vessels in antero-posterior orientation. The PAs and the right superior pulmonary vein are opacified (arrows). Right lower, left upper, and left lower pulmonary veins are not opacified (stars). The calcified bronchial cartilage and coronary calcifications appear black.

A CTPA was ordered to rule out pulmonary embolism, pneumonia or SARS-CoV2 infection. It revealed bilateral central mucoid impaction of the bronchi, sparing only the right upper pulmonary lobe (***Figures 1, 2, 3***). The pulmonary trunk was dilated (***Figure 2***). Only the right upper pulmonary vein was opacified (***Figures 1***, ***4***), suggesting that the other pulmonary veins were not responsible for venous return to the left atrium. Furthermore, the left upper and lower lobes were, respectively, partially and completely collapsed. The right lower lobe suffered aerated collapse due to complete proximal bronchial mucus plugging.The CTPA was negative for pulmonary embolism, pneumonia, or SARS-CoV2.

The patient was unresponsive to oxygen therapy and, after considering the CTPA findings, a decision of therapeutic de-escalation was taken. The patient expired shortly after.

## Discussion

HPV occurs in conditions involving ventilation/perfusion (V/Q) mismatch [1]. The PAs will vasoconstrict, diverting blood away from poorly ventilated to well-ventilated lung segments, where oxygen exchange occurs more readily [1]. By vasoconstricting, local PA resistance increases, promoting blood flow to areas where other arteries are dilated and pulmonary capillaries have been recruited [2]. This mechanism is significant in chronic lung diseases and in acute causes of V/Q mismatch such as asthma, pneumonia, pulmonary embolism, pulmonary edema, or, in this case, intraluminal bronchial obstruction [2]. Vasoconstriction occurs after sensing low O2 partial pressure by an unknown mechanism [2]. The HPV occurs in two phases, the first initiating in seconds, reaching its maximum vascular resistance at 15 minutes [2]. The second phase, occurring if the hypoxic state is maintained for up to an hour, reaches maximum vascular resistance at two hours [2]. After normoxia restoration, the arteries may take hours to relax [2].

Multilobar mucoid impactions are a rare cause of HPV. Occlusive mucoid impaction within the left mainstem and right intermediate bronchus caused reduced ventilatory ability of the patient’s lungs, near complete collapse of the left lower pulmonary lobe, and aerated collapse of the left upper and right lower lobes. We speculate that decreased oxygen partial pressure within these lobes induced vasoconstriction of the corresponding PAs, reducing pulmonary vasculature blood shunting. The increased pulmonary trunk diameter of 38.9 mm (normal being ≤27 mm in females) and dilated right ventricle likely resulted from vasoconstriction-induced severe PA hypertension [3,4]. Lack of contrast entering the left atrium from all but the right superior pulmonary vein suggests a proximal occlusive event, which we believe results from PA vasoconstriction (***Figures 3***, ***4***).

## Conclusion

Hypoxic pulmonary vasoconstriction aims to maintain V/Q homeostasis. Sometimes, the vasoconstriction causes acute PA hypertension and, subsequently, rapid fatal cardiopulmonary failure. This case demonstrates the unusual imagery-physiology correlation of PA vasoconstriction that can normally not be detected by CTPA.
